# Effects of Attribute Affirmation and Achievement Goals on High School Students' Motivation

**DOI:** 10.3389/fpsyg.2021.661668

**Published:** 2021-08-24

**Authors:** Cheng-Hong Liu, Po-Sheng Huang, Xian-Rui Yin, Fa-Chung Chiu

**Affiliations:** ^1^Department of Educational Psychology and Counseling, National Tsing Hua University, Hsinchu, Taiwan; ^2^Graduate Institute of Digital Learning and Education, National Taiwan University of Science and Technology, Taipei City, Taiwan; ^3^Institute for Research Excellence in Learning Sciences, National Taiwan Normal University, Taipei City, Taiwan; ^4^Department of Counseling Psychology, Chinese Culture University, Taipei City, Taiwan

**Keywords:** self-affirmation, achievement goals, attribute affirmation, motivation, challenging tasks

## Abstract

Researchers have suggested that receiving attribute affirmation (AA) may increase the motivation of students to confront a challenge. However, we posited that to determine whether AA increases the motivation of students to confront a challenging task, we must consider dispositional achievement goals of the students. The participants were 171 junior-high-school students, randomly assigned to an AA or no affirmation condition. The results showed that AA enhanced the tendency to confront a challenging task for students who endorsed low mastery-approach goals (MAGs) and low performance-approach goals (PAGs) simultaneously (*b* = 0.5, *p* = 0.015). The effect was mainly mediated by the increasing state performance-approach goals (SPAGs) in confronting the task (indirect effect = 0.21, 95% CI = 0.04–0.49); however, being attribute-affirmed decreased the tendency to confront the challenging task for students adopting a dominant PAG orientation (*b* = −0.76, *p* = 0.049). In addition, for students adopting a dominant MAG orientation or adopting high MAGs and high PAGs simultaneously, no difference was noted in the tendency to confront the task between participants in the control and attribute-affirmed conditions.

## Introduction

Students tend to avoid overwhelming unfamiliar tasks. Teachers must use strategies to stimulate students to actively participate in these tasks. One of the common strategies adopted by teachers is to offer affirmation or praise regarding the attributes of the students when presenting challenges to them (Brophy, 1981; Partin et al., 2010). However, it is uncertain whether attribute affirmation (AA) would actually increase student motivation to overcome challenging tasks. To understand the effect of AA, we hypothesized that students' dispositional achievement goals, which were closely related to the students' motivation to confront challenging tasks (Grant and Dweck, 2003), must be considered.

### AA and Motivation to Confront Challenging Tasks

Trying to resolve challenging tasks may result in failure, and failure threatens the self-worth of the students and may trigger avoidant tendencies (Covington, 1984). This could be further explained using the self-affirmation theory, which proposes that people are motivated to maintain a global image of self-integrity and perception of oneself as a good, competent, moral, and adaptive person. When people are faced with information threatening their self-integrity, they may avoid such information to protect their integrity (Steele, 1988; Sherman and Cohen, 2006; Cohen and Sherman, 2014). In other words, tendencies of students to avoid a challenging task can be seen as a self-protective response. However, according to the theory, if students have opportunities to receive a self-affirmation intervention when facing a challenging task, which is an act that manifests the adequacy of an individual and affirms their sense of global self-integrity, such would increase their self-integrity, reduce the perceived threat of the task, decrease self-protective defensive responses, and increase motivation (Vohs et al., 2013).

Attribute affirmation is one of the popular self-affirmation techniques used in educational settings. The AA intervention is an activity that provides the opportunity to affirm positive qualities of the self. For example, Steele et al. (1993) implemented the AA by giving the participants positive feedback on their personalities (refer to also Koole and van Knippenberg, 2007). Liu and Huang (2019, Experiment 2) informed junior-high-school students in the AA group that they had higher scores on personality than 85% of the other students, after they completed the personality questionnaire, which indicated that their personality is more positive than others. In brief, AA may be a feasible approach for inspiring students to face challenging tasks.

However, it has been controversial in past studies whether affirming attributes of the students could increase their motivation. Specifically, the approach of AA is similar to person praise, an approach often used in educational settings. Person praise refers to offering praise based on the personal qualities of a child, such as abilities (Brummelman et al., 2014). Although conventional wisdom supports the view that praising the traits of a child is beneficial for motivation (Kamins and Dweck, 1999), many researchers have argued that this may harm the motivation of an individual (Henderlong and Lepper, 2002; Henderlong Corpus and Lepper, 2007; Haimovitz and Corpus, 2011; Xing et al., 2018). For example, Mueller and Dweck (1998) discovered that if students are praised for abilities when they succeed in a task, their achievement motivation is reduced when they later fail (refer to also Kamins and Dweck, 1999; Skipper and Douglas, 2012). Pomerantz and Kempner (2013) also found that the more often mothers used personal praise, the more their children avoided the challenges they faced in school. Researchers believe that this may be due to negative self-attribution, as praising students for their attributes or abilities will cause students to direct their attention and attribute their deficiencies to themselves when they fail, thus reducing the motivation to learn (Tangney and Dearing, 2002). Some researchers explain from the entity theory that when students are praised for their attributes or abilities, they tend to form a fixed mindset, believing that their abilities are fixed and will not increase even they invest more effort, and so they are less willing to meet challenges (Pomerantz and Kempner, 2013). Some argue that person praise leads students to believe that praise by others of their abilities is conditional, and that they will only be perceived valuable when they succeed, and unworthy if they fail. Therefore, they will continue to pursue success, hide their weaknesses, and avoid failure to prove that they are valued (Assor et al., 2004). Although such a procedure and research focus of person praise differ from those of the present study (we seek to assess whether AA can increase the motivation of students to face challenging tasks rather than how the motivation of students changes after they are praised for abilities and later fail), these studies highlighted that when individuals face learning tasks, if they are praised for their attributes, their state performance goals might increase and their subsequent motivation might be negatively influenced (Mueller and Dweck, 1998).

This viewpoint has also been supported in some studies investigating the effect of AA on the motivation of people to face challenging tasks. For example, researchers have discovered that AA failed to enhance, or even negatively influence, the motivation of high-school students who were afraid of being laughed at or valued approval of others (Liu et al., 2016; Liu and Huang, 2019). In other words, whether AA is effective in inspiring the learning motivation of the students may be related to whether students possess the characteristic trait of valuing the opinions of others. This trait is related to the construct of performance goals in achievement goal theory, which refers to the states in which an individual tends to gain positive evaluation of others while learning (Elliot and Thrash, 2001). Thus, to understand the influence of AA on the motivation of junior-high-school students to confront challenging tasks, we must consider the dispositional achievement goals of the students.

### AA, Achievement Goals, and Motivation to Confront Challenging Tasks

#### Achievement Goal Types

According to Elliot and Thrash (2001, p. 144), an achievement goal is a cognitive representation that directs the behavior of an individual in a specific direction and involves two main elements, namely, the aim of the behavior and the reason that drives the behavior. For example, in learning situations, individuals exhibit learning behaviors for the aim of performing better than others, and the primary reason may be to gain parental or teacher approval. Elliot and Thrash further propose a hierarchical model that distinguishes achievement goals into four different types based on two dimensions, how competencies are defined or valanced. According to the definition of competence, it is evaluated based on different standards, which are divided into mastery goal (competence is evaluated according to absolute or intrapersonal standard, i.e., according to the own understanding of an individual of the work, or the growth of knowledge or skills) and performance goal (competence is evaluated according to normative standard, that is, the performance of an individual compared with the others). According to the valence of competence, individuals are either focused on obtaining positive results or avoiding attaining negative results, which are further divided into the approach or avoidance goals. Combining the definition and valence of competence, a total of 2 × 2 conceptual categories can be distinguished, namely mastery-approach goals, MAGs (aim to attain mastery and improvement), mastery-avoidance goals (aim to avoid not attaining mastery and improvement), performance-approach goals (PAGs; aim to outperform others or gain positive evaluation of others), and performance-avoidance goals (aim to avoid being worse than others).

Other researchers further divided achievement goals into more categories (e.g., Elliot and Harackiewicz, 1996; Elliot and Church, 1997; Elliot and McGregor, 2001; Elliot and Murayama, 2008; Elliot et al., 2011). However, despite the fact that the categories have increased as research progressed, when discussing the relationship between achievement goals and the related learning outcomes, researchers have mostly focused on examining the effects of MAGs which highlight mastery and improvement of ability, and PAGs which focus on outperforming others (e.g., Linnenbrink, 2005; Lee and Kim, 2014; Ikeda et al., 2015). In addition, some researchers have maintained that when considered as dispositions, learners may hold multiple goals simultaneously. For example, learners may hold different levels of MAGs and PAGs, which may simultaneously affect the learning outcomes of the learner (Linnenbrink, 2005; Levy-Tossman et al., 2007). Therefore, in the present study, when discussing the effect of AA on the motivation of students to confront challenging tasks, we also assessed their tendency to hold these two types of achievement goals.

#### Motivation of Learners With Different Achievement Goals to Confront Challenging Tasks

Research has shown that MAGs can consistently predict challenge-seeking motivation (Lee and Kim, 2014; Mouratidis et al., 2018). The more the individuals with MAGs focus on their growth and gains in their learning task, the more they are willing to seek task challenges that may improve their abilities. Otherwise, individuals with mastery goals hold the incremental belief that the ability of an individual is malleable and that difficulty can be overcome by effort. Therefore, they are willing to seek challenges that will foster learning (Dweck and Leggett, 1988). In addition, researchers have demonstrated that when students leaned toward having mastery goals, their related learning outcomes were more adaptive, whether they exhibited a dominant MAG orientation (i.e., possessing a high level of MAGs but low PAGs) or a combination of MAG and PAG orientation. This indicates that when students are concerned about learning from tasks to increase their abilities, no matter whether they care about receiving positive opinions from others regarding their abilities, they have ample motivation to accept the challenge of learning tasks (Levy-Tossman et al., 2007).

However, if students have a dominant PAG orientation (i.e., possessing a high level of performance-approach but low MAGs), the research results may be inconsistent. That is, students with a dominant PAG orientation are motivated to obtain the positive opinions of others regarding their ability in learning tasks (Elliot and Thrash, 2001). Such motivation does not necessarily enhance their tendency to confront challenging learning tasks. Previous research did support this argument that PAGs are not associated with the challenge seeking of the students (Lee and Kim, 2014; Mouratidis et al., 2018). Those with PAGs place more importance on performing better than others or receiving positive evaluations from others and therefore tend to avoid challenges that they are not sure of success in order not to be threatened by the possible failure of the task. Otherwise, individuals with performance goals hold the entity belief, that the ability of an individual is fixed, and difficulty cannot be overcome with effort. Therefore, they will avoid challenges and appear less persistent when encountering any difficulty (Dweck and Leggett, 1988). In addition, compared with students who endorse low MAGs and low PAGs simultaneously, students who have a dominant PAG orientation overall exhibit a more adaptive outcome (Levy-Tossman et al., 2007). That is, for students simultaneously adopting low MAGs and low PAGs, they may lack the motivation to face challenging tasks the most.

#### The Influence of AA on the Motivations of Learners With Different Achievement Goals to Face Challenging Tasks

We posited that to determine whether AA increases the motivation of students to confront a challenging task, we must consider dispositional achievement goals of the students. Specifically, when students hold MAGs, whether they exhibit a dominant MAG orientation or a combination of MAG and PAG orientation, competence mastery and learning are the primary motivation for them to engage in learning behaviors (Elliot and McGregor, 2001). To attain mastery and improvement, they are willing to accept the challenge of learning tasks (Lee and Kim, 2014; Mouratidis et al., 2018). Although AA can increase the self-integrity of students, reduce the threat of challenging tasks (Vohs et al., 2013), and even increase their state PAGs (Mueller and Dweck, 1998), because these changes may be irrelevant to the primary motivation driving them to accept challenge, we hypothesized that AA is less beneficial to the motivation of students in facing challenging tasks.

Second, for students holding a dominant PAG orientation, the main motivation for them to engage in learning behaviors may be the desire to outperform others and gain positive evaluation about their competence from others (Elliot and McGregor, 2001). Although AA can increase state PAGs of students (Mueller and Dweck, 1998), this desire of gaining positive evaluation has been satisfied *via* AA, which increases their self-integrity. Consequently, they do not need to participate in a challenging task to satisfy this need, and the operation of AA may deprive them of the motivation to actively accept the challenge. Simply put, AA may not be able to increase motivation and may even reduce it for challenging tasks.

Third, students who simultaneously endorse low MAG and low PAGs, they may lack reasons or motivations to engage in learning behaviors or face challenges. However, because AA may increase their self-integrity, reduce the threat of challenging tasks (Vohs et al., 2013), and increase their state PAGs (Mueller and Dweck, 1998), it may give them the motivation to confront challenging tasks. Thus, we hypothesized that AA may increase the tendency of these students to face challenging tasks.

### The Present Study

We aimed to examine the effects of AA and dispositional achievement goals on the motivation of junior-high-school students to confront a challenging learning task and to elucidate possible underlying processes. We hypothesized that the achievement goals (MAGs and PAGs) held by students will moderate the relationship between AA and motivation to confront challenges. Specifically, we predicted that for participants with a dominant MAG orientation or a combination of MAG and PAG orientation, AA cannot help them exhibit a stronger tendency to confront a challenging task. Next, for participants with a dominant PAG orientation, being attribute-affirmed may not be able to increase or it may even decrease their tendency to confront the challenging task. Finally, AA may increase the tendency to confront the challenging task for students possessing low MAGs and low PAGs simultaneously.

In addition, we also hypothesized that the state of PAG is the mediator of the relationship between AA and motivation to confront the challenge. The achievement goals (MAGs and PAGs) held by the students will moderate the mediation of the state of PAG between AA and motivation to confront challenge.

## Methods

### Participants

*A priori* power analysis for multiple regression with seven predictors (AA, MAGs, PAGs, and all the interactions among them) indicated that we needed to have 103 participants to have an acceptable 80% power for detecting a medium-sized effect (*f* ^2^ = 0.15) when employing the traditional 0.05 criterion of statistical significance. By contacting the teachers of seven classes in Western Taiwan, we recruited a total of 180 junior-high-school students of Asian descent. Overall, 95% of the students (*N* = 171; 85 boys, 86 girls) aged 13–14 years (*M* = 13.35, *SD* = 0.48) completed the two-stage study. The participants received a convenience store voucher valued at NT$100 for voluntary participation. They were randomly assigned to an AA (*n* = 86) or a no affirmation control (*n* = 85) condition.

Procedures regarding this study were approved by the Research Ethics Committee of the University in which one of the authors work, and the study was realized in accordance with the APA ethical principles.

### Procedure

In addition to some filler scales, participants in groups of 24–29 first completed the measures for achievement goals, perceived general ability, and general self-esteem (see Measures) on a 6-point scale from 1 (strongly disagree) to 6 (strongly agree). A week later, participants in groups of 20–22 arrived for each session. We informed the participants that we have calculated their personality scores based on their responses on the questionnaire the week before. Similar to the procedure of AA manipulation employed by Koole and van Knippenberg (2007), we informed the participants in the AA condition that they may find that they have some positive characters and some negative characters; however, their personality is fundamentally strong. They were selected together to participate in the next investigation because their personality scores were all higher than that of 85% of the people and that they were with a more positive personality than the other students; however, we informed the participants in the no affirmation control condition that they may find that they have some positive and some negative characteristics and that they were selected together just because of random assignment.

Next, adopting the procedure employed by Liu et al. (2016), we introduced the participants to a novel object-embedding task in which the participants were required to embed several objects of different shapes and sizes closely into a square frame. We informed the participants that the abilities involved in this challenging task are key to success in the endeavors of life and that we intended to test certain students to assess their related abilities. After showing an example of this task, the participants were informed that they would be required to resolve 10 items sequentially in front of other students. Then, in addition to some filler scales, they sequentially completed the measures for self-integrity, tendency to undertake the task, perceived ability on the task, and state PAGs (refer to Measures). Finally, participants were debriefed, thanked for their participation, and dismissed.

### Measures

#### Achievement Goals

The achievement goals of participants were measured with a three-item MAG subscale (e.g., My goal is to learn as much as possible) and a three-item PAG subscale (e.g., My goal is to perform better than the other students) of the achievement goal questionnaire–revised (Elliot and Murayama, 2008). Since we translated this scale into a Chinese version, an exploratory factor analysis (EFA) using the principal component factoring method was conducted on the six items to test the validity of the scale. Inspection of the eigenvalues (greater than 1) and scree plot showed two correlated factors (*r* = 0.53) that underlie these items, accounting for 86% of the variance. All the MAG items loaded above 0.82 and all the PAG items loaded above 0.92 on the intended factors and below 0.32 on the unintended factors. The two subscales possess high internal consistency (α = 0.86, 0.96, respectively).

#### Perceived General Ability

We measured the perceived general ability of the participants with a three-item perceived ability scale (Liu, 2012; e.g., I am confident about my overall ability). An EFA showed that one factor underlies these items, accounting for 70% of the variance (factor loadings > 0.79). The three items possess sufficient internal consistency (α = 0.78).

#### General Self-Esteem

We measured the general self-esteem of the participants with a classical 10-item self-esteem scale (Rosenberg, 1965; e.g., Overall, I am satisfied with myself). An EFA showed that one factor underlies these items accounting for 52% of the variance (factor loadings > 0.37). These items possess sufficient internal consistency (α = 0.89).

#### Self-Integrity

We assessed the self-integrity of the participants with four personality self-integrity items constructed by Liu and Huang (2019; e.g., I have a more positive personality than other students do). These items were included to check the manipulation of AA. An EFA showed one factor underlies these items, accounting for 63% of the variance (factor loadings > 0.68). The items possess sufficient internal consistency (α = 0.80).

#### Tendency to Undertake a Challenging Task

We assessed the tendency of the participants to undertake the challenging task with six items of Liu et al. (2016) (e.g., I am willing to take on the challenge of the object-embedding task). An EFA showed one factor underlies these items accounting for 70% of the variance (factor loadings > 0.79). These items possess high internal consistency (α = 0.91).

#### Perceived Ability on the Challenging Task

We measured the perceived ability of participants on the object-embedding task with three items of Liu (2012) (e.g., I am confident about my ability on the object-embedding task). An EFA showed one factor underlies these items accounting for 67% of the variance (factor loadings > 0.70). These items possess sufficient internal consistency (α = 0.74).

#### State Performance-Approach Goals

We constructed three items regarding SPAGs and used them to assess whether AA increased the **S**PAGs of the participants on the object-embedding task (e.g., the goal of the object-embedding task is to perform better than the other students). These items were adapted from the PAG subscale of Elliot and Murayama (2008). An EFA showed one factor underlies these items accounting for 82% of the variance (factor loadings > 0.90). The items possess high internal consistency (α = 0.89).

## Results

### Preliminary Analyses and Manipulation Check

Table 1 shows the descriptive statistics and correlation matrix for all measures. The object-embedding task was challenging for the participants because they perceived themselves to possess lower ability in performing the task (*M* = 4.1, *SD* = 0.95) than their overall ability (*M* = 4.3, *SD* = 1.1), *t* (170) = 2.47, *p* = 0.014, and *d* = 0.19. Next, participants in the AA condition scored higher on both the four personality self-integrity items and the state PAGs (*M*s = 4.62, 4.02, *SD*s = 0.79, 1.15) than did participants in the non-affirmation condition (*M*s = 4.19, 3.43, *SD*s = 0.8, 1.33), *t*s (169) = 3.52, 3.12, *p*s = 0.001,0.002, and *d*s = 0.54, 0.48. This revealed that AA generally increased the sense of self-integrity and state PAGs of the participants.

**Table 1:** Descriptive statistics, and intercorrelations for all variables.

**Variables**	**1**	**2**	**3**	**4**	**5**	**6**	**7**	**8**	**9**
1. Mastery-approach goals									
2. Performance-approach goals	0.54^**^								
3. Perceived general ability	0.34^**^	0.26^**^							
4. General self-esteem	0.29^**^	0.19^**^	0.78^**^						
5. Attribute affirmation	−0.04	−0.05	−0.01	0.05					
6. Self-integrity	0.24^**^	0.18^**^	0.47^**^	0.41^**^	0.26^**^				
7. Tendencies to undertake the task	0.42^**^	0.17^*^	0.32^**^	0.29^**^	0.07	0.57^**^			
8. Perceived ability on the task	0.16^*^	0.14	0.47^**^	0.46^**^	0.11	0.55^**^	0.58^**^		
9. State performance-approach goals	−0.03	0.13	−0.08	−0.13	0.23^**^	0.28^**^	0.15	0.08	
*M*	4.47	4.37	4.3	4.01	–	4.41	4.53	4.1	4.05
*SD*	1.15	1.25	1.1	0.99	–	0.82	0.96	0.95	1.27

*N = 171*.

* *p < 0.05*,

** *p < 0.01. Attribute affirmation: nonaffirmation = 0, affirmation = 1*.

### Test of Predictions

After the AA condition was dummy-coded (non-affirmation = 0; affirmation = 1) and both MAGs and PAGs were centerd at their means, we tested a moderation model according to Hayes PROCESS Model 3 (Hayes, 2018). In this moderation analysis, we entered MAGs and PAGs as moderators of AA on tendency of students to undertake the task with the effect of self-esteem being controlled. Using the PROCESS program, all analyses included a bias-corrected bootstrap 95% CI based on 5,000 bootstrap samples. The results of an ordinary least squares regression can be found in Table 2 (Model 1).

**Table 2:** Ordinary least squares regression model coefficients.

	**Model 1**		**Model 2**		**Model 3**	
**Outcome**	**Tendencies to undertake** **the task (Motivation)**		**SPAG**		**Tendencies to undertake** **the task (Motivation)**	
**Predictors**	***b***	***t***	***b***	***t***	***b***	***t***
Intercept	3.76	12.25^*^	0.33	0.8	3.65	12.39^*^
SE	0.18	2.61^*^	−0.15	−1.61	0.22	3.25^*^
AA	0.01	0.06	0.58	3.06^*^	0.02	0.11
MAG	0.32	2.86^*^			0.30	2.74^*^
PAG	0.06	0.68			−0.04	−0.41
AA × MAG	0.12	0.84			0.18	1.25
AA × PAG	−0.24	−1.91			−0.08	−0.62
MAG × PAG	−0.13	−1.99^*^			−0.06	−0.96
AA × MAG × PAG	0.23	2.98^*^			0.02	0.21
SPAG					0.12	2.08^*^
SPAG × MAG					−0.01	−0.18
SPAG × PAG					−0.1	−2.15^*^
SPAG × MAG × PAG					0.07	2.95^*^
Model *R*^2^	0.29, *F* (8, 162) = 8.1^*^		0.07, *F* (2, 168) = 6.23^*^		0.37, *F* (12, 158) = 7.66^*^	
AA × MAG × PAG Δ*R*^2^	0.04, *F* (1, 162) = 8.88^*^				0.0001, *F* (1, 158) = 0.04	
SPAG × MAG × PAG Δ*R*^2^					0.03, *F* (1, 158) = 8.68^*^	

*N = 171; SE, mean–centered self-esteem; AA, attribute affirmation; MAG, mean-centered mastery-approach goals; PAG, mean-centered performance-approach goals. SPAG, mean-centered state performance-approach goals*.

* *p < 0.05*.

**Figure 1:**
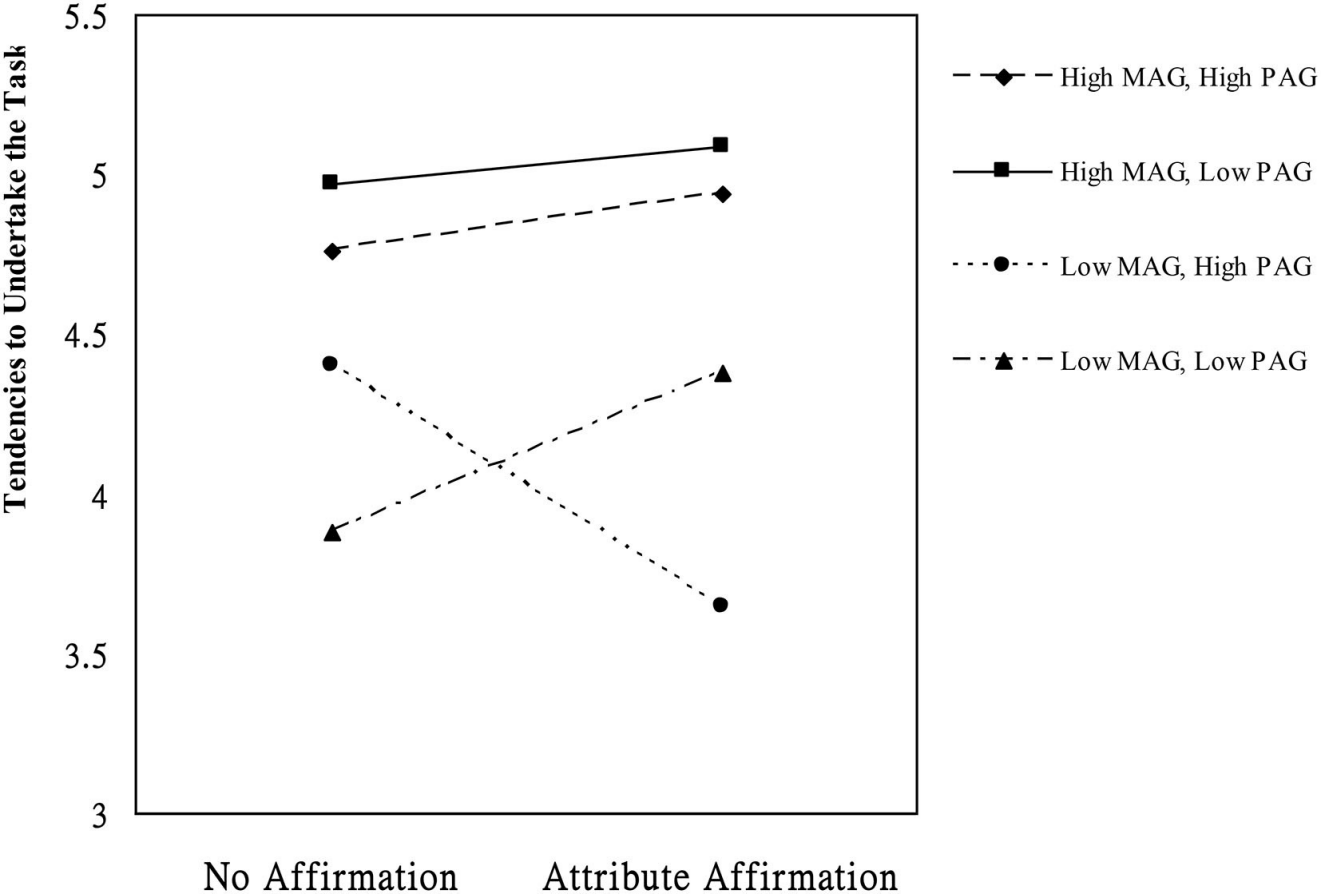
Mean of tendencies to undertake the task as a function of attribute affirmation, mastery-approach goals (MAG), and performance-approach goals (PAG; High = 1 SD above mean, Low = 1 SD below mean).

The results showed that the variance of the tendency to undertake the task accounted for was 29%. The results from Model 1 revealed the AA × MAG × PAG interaction was significant, *b* = 0.23, *t* = 2.98, *p* = 0.003, and Δ*R*^2^ = 0.04. We depicted this interaction graphically in Figure 1, which shows the graph of tendency of the students to undertake the task as a function of AA, MAG, and PAG. Simple slope analysis showed that for students who possessed high MAG and high PAG simultaneously and who possessed high MAG but low PAG simultaneously, no difference was noted in the tendency to confront the challenging task between participants in the control and attribute-affirmed conditions, *b*s = 0.18, 0.11, *t*s = 0.82, 0.36, and *p*s = 0.41, 0.72. However, for students who simultaneously possessed low MAG but high PAG, those in the attribute-affirmed condition exhibited weaker tendency to confront the challenging task relative to those in the control condition, *b* = −0.76, *t* = −1.99, and *p* = 0.049. In addition, for students who possessed low MAG and low PAG simultaneously, being attribute-affirmed enhanced the tendency to confront the challenging task, *b* = 0.5, *t* = 2.47, and *p* = 0.015.

### Mediation Analysis

To test the mediating role of SPAGs underlying the SA × MAG × PAG interaction, a set of regression analyses for testing mediating mechanisms underlying interaction were conducted using Hayes PROCESS Model 19 (Hayes, 2018; as shown in Model 2 and Model 3 in Table 2). First, a regression analysis was performed to examine the effect of AA on SPAGs with the effect of self-esteem being controlled (as shown in Model 2 in Table 2). The results showed that AA increased the SPAGs of the participants, *b* = 0.58, *t* = 3.06, and *p* = 0.002.

Next, a regression analysis (as shown in Model 3 in Table 2) not only included the effects of all the previously constructed regression terms in Model 1 on tendency to undertake the task, but also added parallel terms replacing AA with mean-centered SPAGs to the equation (i.e., SPAG, SPAG × MAG, SPAG × PAG, and SPAG × MAG × PAG). The results showed that the SPAG × MAG × PAG interaction on tendency to undertake the task was significant, *b* = 0.07, *t* = 2.95, and *p* = 0.003. In addition, after this interactive effect was considered, the significant AA × MAG × PAG interaction on tendency to undertake the task, as identified in Model 1, disappeared. In addition, the results revealed a significant indirect effect of the highest order interaction (i.e., a significant moderated mediation effect = 0.043, 95% CI = 0.01 to 0.11), indicating that the moderating effect of MAG and PAG on the relationship between AA and tendency to undertake the task (MODEL 1) is mediated by the effect of AA on SPAG (MODEL 2) and the moderating effect of MAG and PAG on the relationship between SPAG and tendency to undertake the task (MODEL 3). More specifically, the results for the conditional indirect effects showed that the mediation of AA on the tendency of students to undertake the task through SPAG only occurred in participants possessing low MAG and low PAG simultaneously (with a positive indirect effect = 0.21, 95% CI = 0.04 to 0.49). However, among participants holding high MAG and high PAG simultaneously (indirect effect = 0.05, 95% CI = −0.04 to 0.16), holding high MAG but low PAG simultaneously (indirect effect = 0.08, 95% CI = −0.09 to 0.22) and holding low MAG but high PAG simultaneously (indirect effect = 0.11, 95% CI = −0.29 to 0.18), there was no evidence of this process at work.

## Discussion

In the current study, we examined the effects of AA and dispositional achievement goals on motivation of junior-high-school students to confront a challenging task. We used Taiwanese junior-high-school students as participants. Overall, the results were mostly consistent with our predictions. Below we interpret the findings and discuss the implications.

### Effect of AA on the Motivation of Students Depends on Dispositional Achievement Goals

According to self-affirmation theory, the AA intervention may increase motivation of the students to confront challenging tasks. These results did show that for participants possessing low MAG and low PAG simultaneously, those who received positive personality feedback exhibited stronger tendency to confront the challenging task relative to those who received a neutral personality feedback. This reveals that AA would help motivate these students to confront challenging tasks. However, for those exhibiting a dominant PAG orientation, attribute-affirmed participants exhibited a weaker tendency to confront the challenging task relative to non-affirmed participants. This reveals that being attribute-affirmed may decrease the tendency to confront the challenging task for these students. In addition, for participants possessing high MAGs (including those with a dominant MAG orientation or a combination of MAG and PAG orientation), no difference was noted in the tendency to confront the challenging task between participants in the control and attribute-affirmed conditions. It should be noted that the MAGs held by participants can positively influence their motivation to confront challenging task. This reveals that individuals with high MAGs would enhance their motivation to confront the challenge, but AA may not add further benefit for motivating these students to face challenging tasks. Overall, these results indicate that whether the AA intervention increases the motivation of junior-high-school students to confront a challenging task depends on the dispositional achievement goals of the students.

### Why Does AA Only Increase the Motivation of Some Students?

These results demonstrated that AA, in general, increased the SPAGs of the participants. However, the mediation analysis revealed that the mediation of AA on the tendency of students to undertake the task through SPAGs only occurred in participants possessing low MAGs and low PAGs simultaneously but did not occur in participants holding the other three types of goal orientation. We interpret this to mean that for students lacking any motivation when facing learning tasks (possessing low MAGs and low PAGs simultaneously), AA would lead them to exhibit higher SPAGs, which in turn increased their tendency to confront the challenging task. However, for students holding a combination of MAG and PAG orientation or a dominant MAG orientation, even though AA increased their SPAGs, such goals were minimally helpful because this motivation was less relevant to the primary motivation (learning from tasks and increasing abilities) of these students facing a challenging task. In addition, for students holding a dominant PAG orientation, even though AA also increased their SPAGs, the main source of motivation of these students to confront a challenging task came from a quest to maintain the positive opinion of others. This need may have been satisfied prematurely during the AA operation process (in that AA increases self-integrity of the students), so they did not need to participate in the challenging task to satisfy their needs. Therefore, AA did not help increase their tendency to accept the challenging task but rather reduced their tendency to proactively accept the task.

### Theoretical and Practical Implications

This study was the first to integrate self-affirmation theory and achievement goal theory to investigate student motivation; it discovered that if AA is used to increase motivation of the students to face challenging learning tasks, then the original goals and reasons for the students to face the learning tasks must be considered. For learners lacking the motivation to face learning tasks (e.g., neither wanting to learn and improve abilities from the learning task nor desiring to obtain a positive opinion from others through this task), the intervention of AA may provide them with higher SPAGs, thereby increasing their learning motivation. This is consistent with the results of studies on person praise. In addition, for students who cared more about receiving positive opinions about their ability and cared less about learning and improving their abilities through a learning task, the intervention of AA might prematurely satisfy their need in this regard and consequently reduce their motivation when facing learning tasks; however, as for students who cared about learning from a learning task to improve their abilities, no matter whether they also cared about receiving positive opinions from others through the learning task, the AA intervention might have offered limited help in motivating them to face learning tasks. But it is noteworthy to mention that, possessing MAGs itself will enhance individuals to confront challenges. In summary, this study discovered a novel result that when AA is to be used to improve student motivation, the dispositional achievement goals of the students must be considered.

These findings have critical practical implications in the educational setting where teachers often adopt measures related to AA (such as person praise) to encourage students to face challenging tasks. Specifically, these results indicate that if teachers adopt this method to encourage students, they must consider dispositional achievement goals of the students. If students lack motivation while facing challenging tasks (for example, not wanting to learn from tasks or improve their abilities or wanting to receive positive opinions from others), then using measures related to AA to encourage student motivation may be conducive. However, when students tend to care about maintaining positive opinions of others about themselves and care less about learning and improving their abilities, then measures related to AA should be avoided because this application may weaken the motivation of students to confront challenging learning tasks. By contrast, if students face challenging tasks and tend to focus on learning from the task to improve their abilities, then even without measures related to AA, they still exhibit high motivation. Therefore, it is suggested that educators may promote MAGs of students, which can effectively enhance their tendency to confront challenges.

### Limitations and Future Directions

There are several limitations in the current study. First, we only have results from a single study to report. In this experiment, participants were all junior-high-school students, and only one challenging task was used. We suggest that future studies use samples at different stages and adopt different tasks in their experiments to see if the results from the present study are robust. Second, the participants of the present study were mainly Asian students from Taiwan. We suggest that future studies use samples from different cultures to see if the results of the present study apply to different cultures. Third, the achievement goals of this study were obtained through measuring the dispositional achievement goals. However, the achievement goals that the students obtained could be the state achievement goals induced by situational cues or instructions. We suggest that future studies induce state achievement goals of students through manipulating situational cues to clarify whether the effect of AA on the motivation to confront challenges differs due to this manipulation. Fourth, this study only investigated the effects of AA and achievement goals on the motivation to confront challenging tasks. We suggest that future researchers investigate whether AA and achievement goals further influence subsequent performance of the students after influencing their motivation to face challenging tasks.

## Data Availability Statement

The raw data supporting the conclusions of this article will be made available by the authors, without undue reservation.

## Ethics Statement

The studies involving human participants were reviewed and approved by Ethics committee of National Tsing Hua University. Written informed consent to participate in this study was provided by the participants' legal guardian/next of kin.

## Author Contributions

C-HL and P-SH: interpretation of results and write-up. C-HL, P-SH, X-RY, and F-CC: data analysis and data collection. C-HL and P-SH: study conceptualization.

## Conflict of Interest

The authors declare that the research was conducted in the absence of any commercial or financial relationships that could be construed as a potential conflict of interest.

## Publisher's Note

All claims expressed in this article are solely those of the authors and do not necessarily represent those of their affiliated organizations, or those of the publisher, the editors and the reviewers. Any product that may be evaluated in this article, or claim that may be made by its manufacturer, is not guaranteed or endorsed by the publisher.
